# Explore the efficacy of microwave ablation combined with fruquintinib and tislelizumab in the treatment of metastatic colorectal cancer

**DOI:** 10.3389/fonc.2026.1728876

**Published:** 2026-02-24

**Authors:** Wei Qian, Huatang Zhang, Yanhua Mo, Chaoyang Wu, Juntong Liu, Yuyi Pen, Cantu Fang, Jincheng Meng

**Affiliations:** Zhongshan Hospital of Traditional Chinese Medicine Affiliated to Guangzhou University of Chinese Medicine, Zhongshan, Guangdong, China

**Keywords:** fruquintinib, immunotherapy, metastatic colorectal cancer, microwave ablation, tislelizumab

## Abstract

**Background:**

Currently, the prognosis for metastatic colorectal cancer (mCRC) remains unfavorable. However, as advancements in local and systemic treatment modalities progress, therapeutic strategies for mCRC are becoming increasingly varied. For patients presenting with a limited number of metastases, the integration of radical local treatment with systemic therapy holds promise for achieving sustained tumor control. This study seeks to investigate the efficacy and safety of combining microwave ablation (MWA) with fruquintinib and tislelizumab in patients with mCRC.

**Methods:**

Between March 2022 and September 2022, a screening process was conducted on patients with advanced colorectal cancer and liver metastases who had previously undergone radical colon cancer surgery at our institution. Eligible patients commenced a combined treatment regimen of fruquintinib (5 mg, administered daily from day 1 to day 14) and tislelizumab (200 mg, administered on day 1 every three weeks) within one week following the completion of MWA. Post-surgery, 21 patients received the combination of fruquintinib, a molecular-targeted therapy, and tislelizumab immunotherapy, whereas 34 patients were administered tislelizumab in conjunction with a placebo as the molecular-targeted treatment. The study evaluated all patients’ progression-free survival (PFS), objective response rate (ORR), disease control rate (DCR), and three-year overall survival (OS).

**Results:**

In the study, the median PFS was 13.2 months for the experimental group compared to 7.35 months for the control group, with 1-year PFS rates of 66.7% and 23.5%, respectively. Tislelizumab monotherapy emerged as an independent risk factor for tumor recurrence, with the experimental group exhibiting a 0.184-fold reduction in recurrence risk relative to the control group (P = 0.036). Multivariate Cox regression analysis further identified elevated carcinoembryonic antigen levels and the number of liver metastases as significant risk factors for tumor recurrence. Specifically, patients with normal carcinoembryonic antigen levels demonstrated a 0.12-fold lower recurrence risk compared to those with elevated levels (P = 0.0002), while patients with a single liver metastasis had a 0.208-fold lower recurrence risk than those with multiple metastases (P = 0.0003). The ORR were 72.5% in the experimental group and 58.8% in the control group, indicating a significantly higher ORR in the experimental group. Additionally, the experimental group exhibited superior OS compared to the control group, with both groups achieving an OS exceeding 20 months.

**Conclusion:**

This study aims to provide novel clinical evidence supporting the integration of MWA with PD-1 inhibitors and anti-angiogenic agents in the systemic management of mCRC. It is anticipated that these findings will expand therapeutic options available to patients.

**Clinical Trial Registration:**

https://www.chictr.org.cn/showproj.html?proj=140822, identifier ChiCTR 2200058323 (China Clinical Trial Center).

## Introduction

Colorectal cancer is one of the predominant malignancies of the digestive tract in China, both in terms of incidence and mortality (1).Epidemiological data reveal that approximately 20%-30% of patients are diagnosed with metastatic disease at the initial presentation, imposing a significant disease burden (2).While systemic treatments for metastatic colorectal cancer (mCRC)—including chemotherapy, targeted therapy, and immunotherapy—have made substantial advancements (3), increasing the median overall survival of patients with advanced disease from approximately 6 months in the era of chemotherapy alone to over 30 months with current combination targeted therapies (4), achieving long-term survival remains a formidable challenge. Numerous clinical studies indicate that the global 5-year survival rate for mCRC is approximately 10-15%, with similar statistics reported in certain cancer registry regions in China (5). This suggests that current systemic treatment strategies continue to encounter significant obstacles in overcoming tumor heterogeneity and preventing drug resistance.

In the context of therapeutic challenges, the significance of local treatments for “oligometastatic” disease has become increasingly recognized (6). Among these treatments, microwave ablation, a thermal ablation technique, has shown promising results in managing oligometastatic lesions in the liver and other locations due to its unique advantages (7).Large-scale clinical studies and meta-analyses have demonstrated that for unresectable colorectal liver metastases, microwave ablation achieves an initial technical success rate of 88% to 95%, with local tumor control rates exceeding 85% across various centers. Importantly (8), when combined with effective systemic therapy, microwave ablation provides significant survival benefits for carefully selected patients with oligometastatic disease. Some studies have reported 5-year survival rates increasing to 40% to 50%, which is a substantial improvement over historical controls receiving only systemic therapy (9). Additionally, anti-angiogenic therapy, which focuses on modulating the tumor microvascular environment, has emerged as a crucial component in comprehensive cancer treatment (10).These agents facilitate the normalization of vascular architecture and function, enhance local tumor perfusion, and mitigate hypoxia and acidosis, thereby increasing sensitivity to radiotherapy and specific chemotherapeutic agents. Simultaneously, anti-angiogenic drugs exhibit various immunomodulatory effects, working synergistically with immune checkpoint inhibitors to collectively counteract immunosuppressive microenvironments and augment antitumor immune responses (11). Motivated by these findings, this study compiles and analyzes clinical patient data to evaluate the prognosis and efficacy of integrating targeted therapy and immunotherapy with microwave ablation (MWA).

## Materials and methods

### Exclusion criteria

Patients enrolled at our hospital from March 1, 2022, to September 30, 2022

Inclusion Criteria

1. Patients aged 18–75 years (inclusive);

2. Pathologically confirmed stage IV primary colorectal cancer;

3. Metastasis to one or multiple organs (oligometastatic), with metastatic lesions deemed suitable for microwave ablation radiotherapy by investigator assessment;

4. Previous failure of at least two lines of standard therapy (based on fluorouracil, oxaliplatin, irinotecan, bevacizumab, or cetuximab);

Note: Adjuvant/neoadjuvant therapy is permitted. If recurrence occurs during or within 6 months after adjuvant/neoadjuvant therapy, such therapy is considered first-line treatment for advanced disease;

5. At least one extracranial measurable lesion meeting RECIST 1.1 criteria;

6. If the subject underwent surgery, they must be fully recovered from the toxicity and complications of the surgical intervention prior to treatment initiation before consideration for enrollment;

7. ECOG performance status: 0–1;

8. Expected survival ≥12 weeks;

9. Organ function meets the following requirements (no use of blood components or cell growth factors within 2 weeks prior to enrollment):

• Bone marrow function: Neutrophil count ≥ 1.5 × 10^9^/L, white blood cell count ≥ 4.0 × 10^9^/L, platelets ≥ 100 × 10^9^/L, hemoglobin ≥ 90 g/L;
• Liver: Serum total bilirubin (TBIL) ≤ 1.5 times ULN. When serum total bilirubin exceeds 1.5 times ULN, direct bilirubin must ≤ ULN. ALT and AST ≤ 2.5 times ULN (expanded to 5 times ULN for patients with liver metastases);
• Renal: Blood urea nitrogen (BUN) and creatinine (Cr) ≤ 1.5×ULN (with creatinine clearance (CCr) ≥ 50 mL/min);
• Cardiac: Normal cardiac function with left ventricular ejection fraction (LVEF) ≥ 50%;

Coagulation: INR ≤ 1.5×ULN, APTT ≤ 1.5×ULN.

10. Male or female patients of reproductive potential must voluntarily use effective contraception during the study and for 6 months after the last study dose, such as dual-barrier contraception, condoms, oral or injectable contraceptives, intrauterine devices, etc. All female patients will be considered reproductive age unless they have reached natural menopause, undergone artificial menopause, or undergone sterilization procedures (e.g., hysterectomy, bilateral adnexectomy, or ovarian irradiation). Otherwise, female patients must demonstrate non-pregnancy via serum testing (within 7 days prior to study entry) and must be non-lactating.

Exclusion Criteria

1. Previous treatment with anti-PD-1/PD-L1 immunotherapy or other investigational immunotherapy drugs;
2. Patients with severe autoimmune diseases: active inflammatory bowel disease (including Crohn’s disease, ulcerative colitis), rheumatoid arthritis, scleroderma, systemic lupus erythematosus, autoimmune vasculitis (e.g., Wegener’s granulomatosis);
3. Symptomatic interstitial lung disease or active infectious/non-infectious pneumonia;
4. Risk factors for intestinal perforation: active diverticulitis, intra-abdominal abscess, gastrointestinal (GI) obstruction, abdominal cancer, or other known risk factors for intestinal perforation;
5. Patients who have undergone surgery must wait until complete wound healing before considering enrollment;
6. History of other malignancies; however, cured localized tumors such as basal cell carcinoma of the skin, squamous cell carcinoma of the skin, superficial bladder cancer, carcinoma *in situ* of the prostate, carcinoma *in situ* of the cervix, or carcinoma *in situ* of the breast are permissible;
7. Patients scheduled for or who have previously undergone organ or allogeneic bone marrow transplantation;
8. Patients with clinically symptomatic moderate to severe ascites requiring therapeutic paracentesis or drainage, or Child-Pugh score >2 (excluding those with imaging-detected minimal ascites without clinical symptoms); uncontrolled or moderate-to-large pleural effusion or pericardial effusion;
9. History of gastrointestinal bleeding within 6 months prior to study treatment initiation, or confirmed risk factors for gastrointestinal bleeding, such as: high-risk or severe esophageal/gastric varices, active localized peptic ulcer lesions, persistent positive fecal occult blood test (FOBT) (if FOBT is positive at baseline, retest is permitted; if still positive after retest, an upper endoscopy (EGD) is required; if EGD indicates esophageal or gastric varices with bleeding risk, the patient is ineligible);
10. History of abdominal fistula, gastrointestinal perforation, or abdominal abscess within 6 months prior to study treatment initiation;
11. Known hereditary or acquired bleeding disorders (e.g., coagulation disorders) or thrombotic tendencies, such as hemophilia; currently receiving or having recently received (within 10 days prior to study treatment initiation) full-dose oral or injectable anticoagulants or thrombolytic agents for therapeutic purposes (low-dose aspirin or low molecular weight heparin for prophylaxis is permitted);
12. Current or recent (within 10 days prior to study treatment initiation) use of aspirin (>325 mg/day [maximum antiplatelet dose]), dipyridamole, ticlopidine, clopidogrel (≥75 mg), or cilostazol;
13. Patients with active infection, heart failure, myocardial infarction within the past 6 months, unstable angina, or unstable arrhythmia;
14. Physical examination findings or clinical laboratory results that may interfere with study outcomes or increase the risk of treatment-related complications, or other uncontrollable medical conditions;
15. Breastfeeding or pregnant women;
16. Congenital or acquired immunodeficiency disorders, including human immunodeficiency virus (HIV), or history of organ transplantation or allogeneic stem cell transplantation;
17. Patients with psychiatric disorders, substance abuse, or social issues affecting compliance, as determined by physician review;
18. Known active infections, including active pulmonary tuberculosis; however, patients with hepatitis B virus (HBV) or hepatitis C virus (HCV) infection may be enrolled if stable after antiviral therapy;
19. Patients who received live vaccines within 30 days prior to enrollment (Note: Seasonal influenza vaccines administered via injection are typically inactivated vaccines and are therefore permitted; intranasal formulations are usually live attenuated vaccines and are not permitted);
20. Uncontrolled cardiac clinical symptoms or diseases, such as: (1) NYHA Class II or higher heart failure (see Appendix 5) or echocardiography showing LVEF <50%; (2) unstable angina; (3) myocardial infarction within 1 year prior to study initiation; (4) Clinically significant supraventricular or ventricular arrhythmias requiring treatment or intervention; (5) QTc > 450 ms (males); QTc > 470 ms (females) (QTc interval calculated using Fridericia’s formula; if QTc is abnormal, measure three consecutive times at 2-minute intervals and take the average);
21. Hypertension uncontrolled despite antihypertensive therapy (systolic BP ≥140 mmHg or diastolic BP ≥90 mmHg) (based on the mean of ≥2 BP measurements); achievement of these parameters via antihypertensive therapy is permitted; history of hypertensive crisis or hypertensive encephalopathy;
22. Major vascular events within 6 months prior to study treatment initiation (e.g., aortic aneurysm requiring surgical repair or recent peripheral arterial thrombosis);
23. Severe, unhealed, or open wounds; active ulcers; or untreated fractures;
24. Major surgical procedures within 4 weeks prior to study treatment initiation (except for diagnostic procedures) or anticipated major surgery during the study period;
25. Inability to swallow tablets, malabsorption syndrome, or any condition affecting gastrointestinal absorption;
26. Evidence of intra-abdominal gas accumulation not explained by puncture or recent surgery;
27. Metastatic disease involving major airways or blood vessels (e.g., complete occlusion of the portal vein trunk or vena cava due to tumor invasion requires exclusion; the portal vein trunk refers to the confluence of the splenic vein and superior mesenteric vein, as well as the bifurcation of the portal vein into left and right branches) or large central mediastinal masses located near the carina (<30 mm distance);
28. History of hepatic encephalopathy;
29. Current interstitial pneumonia or interstitial lung disease, or prior history of interstitial pneumonia or interstitial lung disease requiring corticosteroid treatment, or other pulmonary fibrosis, organizing pneumonia (e.g., obliterative bronchiolitis), pneumoconiosis, drug-induced pneumonia, idiopathic pneumonia, or evidence of active pneumonia on screening chest computed tomography (CT) scan, or severe pulmonary impairment. Radiation pneumonitis in the radiation field is permitted. Active tuberculosis;
30. Active autoimmune disease or history of autoimmune disease with potential for recurrence (including but not limited to: autoimmune hepatitis, interstitial pneumonia, uveitis, enteritis, hypophysitis, vasculitis, nephritis, hyperthyroidism, hypothyroidism [subjects controllable only with hormone replacement therapy may be included]); Subjects with skin conditions not requiring systemic treatment (e.g., vitiligo, psoriasis, alopecia), controlled type 1 diabetes managed with insulin, or childhood asthma in complete remission requiring no adult intervention may be included; asthma patients requiring bronchodilator intervention are excluded;
31. Use of immunosuppressive agents or systemic corticosteroids for immunosuppression within 14 days prior to study treatment initiation (dose >10 mg/day prednisone or equivalent);
32. Known history of severe hypersensitivity to any monoclonal antibody or anti-angiogenic targeted therapy;
33. Severe infection within 4 weeks prior to study treatment initiation, including but not limited to hospitalization due to infection, bacteremia, or severe pneumonia complications; therapeutic antibiotic administration (oral or intravenous) within 2 weeks prior to study treatment initiation (patients receiving prophylactic antibiotics, e.g., for urinary tract infection prevention or chronic obstructive pulmonary disease exacerbation prevention, are eligible for study participation);
34. Other factors judged by the investigator to potentially affect study outcomes or necessitate premature study termination, such as alcohol abuse, substance abuse, other serious medical conditions (including psychiatric disorders) requiring concomitant treatment, severe laboratory abnormalities, or family/social factors compromising patient safety.

### Random grouping method

In this study, a simple random number table method was employed to allocate research subjects meeting the inclusion criteria into groups, ensuring that the baseline data for both the experimental and control groups were balanced and comparable. The specific implementation process was as follows:

1. Inclusion and Numbering: Research subjects were continuously recruited based on predetermined inclusion and exclusion criteria (refer to the aforementioned criteria for details). Each subject was assigned a unique consecutive number according to the order of recruitment (numbering range: 1 to N, where N = 80, representing the total sample size ultimately included). Concurrently, baseline characteristic data for all subjects were collected, including key confounding factors such as age and baseline gender indicators, to facilitate subsequent tests for group balance.
2. Generation of Random Sequence: The SPSS 25.0 statistical software was utilized to generate a random number sequence, with the seed number set to 202008. The generated random numbers were integers ranging from 0 to 99, ensuring that each sample number corresponded to a unique random number without repetition or omission.
3. Grouping Rules and Implementation: Clear grouping rules were established. Following the arrangement of random numbers in ascending order, the first 50% of the samples were allocated to the experimental group, while the remaining 50% were assigned to the control group. This grouping process was independently conducted by a third-party researcher who was not involved in sample recruitment, data collection, or intervention implementation, thereby minimizing selection bias. The results of the grouping were preserved in sealed and numbered envelopes. The intervention implementer only opened these envelopes to confirm the group assignments after the collection of baseline information for all samples, thus ensuring “allocation concealment.”
4. This study employed a double-blind design, ensuring that neither the participants nor the researchers were aware of the group assignments. An independent third-party institution was tasked with preparing the intervention drugs for both the experimental and control groups. A professional statistician generated the random allocation sequence, and the “correspondence between random numbers and groups” was securely stored in a confidential file at the third-party institution. The research team remained blinded to this coding information until the conclusion of the study, when the data were locked and the blind was broken. Throughout the implementation of the intervention measures, the integrity of the blinding method was maintained through a standardized operational process and uniform intervention materials.
5. Verification of Grouping Balance: Following the completion of the grouping process, a statistical analysis was performed to assess the baseline characteristics of the two groups of research subjects. Measurement data were presented as “mean ± standard deviation (x ± s),” and comparisons between groups were conducted using the independent sample t-test. Categorical data were expressed as “number of cases,” with comparisons between groups made using the X^2^ test. For ordinal data, the rank-sum test was employed. The analysis revealed no statistically significant differences between the two groups in key indicators, such as age and baseline gender values (P > 0.05; refer to **Table 1** for details). These findings indicate that the random allocation process in this study successfully ensured the comparability of the two groups.

**Table 1:** Baseline characteristics of patient.

Variable	Experimental group	Control group	t	P
Gender				
Male	14	22	0.148	0.882
Female	7	12		
Age (years), Mean ± sd	43.62 ± 11.58	43.61 ± 12.27	0.004	0.992
AFP, Median (Min, Max)	23.8 (4.2,720.3)	19.2 (3.8,930.1)	1.24	0.22
CEA, Median (Min, Max)	16.8 (2.3,186.4)	24.7 (2.8,235.4)	-1.85	0.071
CA19-9, Median (Min, Max)	53.2 (18.6,92.3)	54.6 (15.3,94.8)	-0.42	0.68
PIC grade, Median (Min, Max)	1 (0,2)	1 (0,2)	0.18	0.86
KPS grade, Median (Min, Max)	80 (60,100)	80 (60,100)	0.15	0.88
The maximum size of the primary tumor, Median (Min, Max)	3.89 (1.85, 8.23)	4.46 (1.72, 6.12)	-1.62	0.11
MSI				
MSI-H	10	17	0.74	0.39
MSI-L	0	1		
MSS	11	16		
RAS				
RAS gene wild-type	10	17	0.32	0.79
RAS gene mutation	11	17		
Primary lesion site				
Left hemicolon	5	9	-0.06	0.07
Right hemicolon	13	17		
Rectum	3	8		
Number of metastatic tumors				
Single	18	26	0.83	0.41
Multiple	3	8		
Hepatitis				
Yes	17	28	-0.16	0.87
No	4	6		
Ascites				
Yes	1	1	0.47	0.64
No	20	33		

AFP, alpha-fetal protein;CEA, carcinoembryonic antigen; CA19-9, tumor marker 19-9; PIC, peritoneal Cancer Index; ECOG, easternCooperativeOncology Group; KPS, karnofsky.

### Treatment method

MWA is performed using Merri Medical Equipment System’s ultrasound guidance and Toshiba Medical Aquilion One computed tomography scanner. All MWA procedures utilize the Amate C-3C Microwave Ablation System (Vison Medical). This device operates at a frequency of 2450 ± 50 MHz with a continuous wave output power range of 120W, equipped with an ablation antenna measuring 18 cm in length and 2 mm in diameter. Ablation mode, power, and duration parameters are selected based on lesion size. Standard ablation power ranges from 50-70W for 8–11 minutes, with ablation coverage extending at least 1–2 cm beyond tumor margins. All procedures are performed by experienced senior physicians.

Postoperatively, patients are randomized into experimental and control groups. The experimental group initiated targeted and immunotherapy with fruquintinib combined with tislelizumab within one week after microwave ablation. Fruquintinib was administered orally at 5 mg once daily for two weeks, followed by a one-week break, in three-week cycles. Tislelizumab was administered intravenously at 200 mg on the first day of each cycle, in three-week cycles. The control group initiated molecular targeted therapy with tislelizumab combined with placebo within one week after microwave ablation. The placebo, after being discreetly processed by an external institution, matches the study drug in appearance, taste, and route of administration. Assessment occurred after three consecutive treatment cycles post-surgery, continuing until disease progression or voluntary withdrawal of informed consent during the treatment period. According to the Common Terminology Criteria for Adverse Events (CTCAE), treatment with the combination therapy will be suspended for Grade 3 or 4 adverse reactions including hematologic toxicity, skin toxicity, gastrointestinal toxicity, hypertension, or abnormal liver function. Treatment will resume after symptom resolution or upon the attending physician’s assessment of whether to continue the original treatment plan.

### Follow-up and evaluation

This study conducted a 3-year follow-up with a cutoff date of September 30, 2025. The primary endpoint was progression-free survival (PFS) assessed according to RECIST v.1.1. PFS was defined as the time from the date of treatment initiation to the first occurrence of disease progression or death from any cause, whichever occurred first. Liver contrast-enhanced CT or magnetic resonance imaging (MRI) was performed approximately one month after microwave ablation combined with medication. CT or MRI scans were repeated monthly after completing three treatment cycles, followed by quarterly intervals thereafter. For subjects without disease progression during the trial, PFS was defined as the date of the last confirmed progression-free event. In this study, recurrence was defined as including local progression and distant metastasis. Local progression referred to the emergence of any new tumor lesions in the liver, while distant metastasis indicated the presence of portal vein invasion, lymph node metastasis, or tumor lesions in other organs. Secondary endpoints included objective response rate (ORR), disease control rate (DCR), overall survival (OS), and patient quality of life, all assessed according to RECIST v.1.1.Radiologic response assessments in this study were conducted according to RECIST 1.1 criteria by two independent radiologists who were blinded to the treatment allocation and clinical outcomes of the patients.

### Data analysis

Statistical analysis was performed using SPSS 25.0 software. For baseline quantitative data, mean and standard deviation were used to describe distribution characteristics when data followed a normal distribution. Independent samples t-tests were used to compare differences between groups. For non-normally distributed data, median and range were used to describe distribution, and the Wilcoxon signed-rank test was employed for intergroup comparisons. Categorical data comparisons utilized chi-square tests or Fisher’s exact probability test.

## Results

### Patient baseline

In this study, 80 patients with metastatic colorectal cancer undergoing MWA were screened based on inclusion criteria. After excluding 25 cases (17 due to insufficient drug cycles, 8 lost to follow-up), A total of 55 patients were ultimately enrolled. Among them, 21 patients in the experimental group received targeted immunotherapy after microwave ablation (MWA), while 34 patients in the control group received placebo combined with targeted therapy after MWA. There were no statistically significant differences between the two groups in terms of gender, age, MSI,RAS,primary lesion site, alpha-fetoprotein (AFP), carcinoembryonic antigen (CEA), CA19–9 marker, tumor burden (TB), primary tumor maximum size, tumor number, hepatitis status, ascites presence, peritoneal cancer index (PCI), ECOG performance status, and Child-Pugh classification (P>0.05). This indicates balanced and comparable baseline characteristics between groups (see **Table 1**).

### PFS comparison

After completing three treatment cycles, both the experimental and control groups underwent one-year follow-up to assess PFS. The median PFS in the control group was 7.35 months. Compared to the control group, the intervention strategies in the experimental group significantly delayed disease progression and extended patients’ progression-free survival. Moreover, some patients in the experimental group still maintained a progression - free state at 15 months, indicating better clinical benefits.

**Figure 1:**
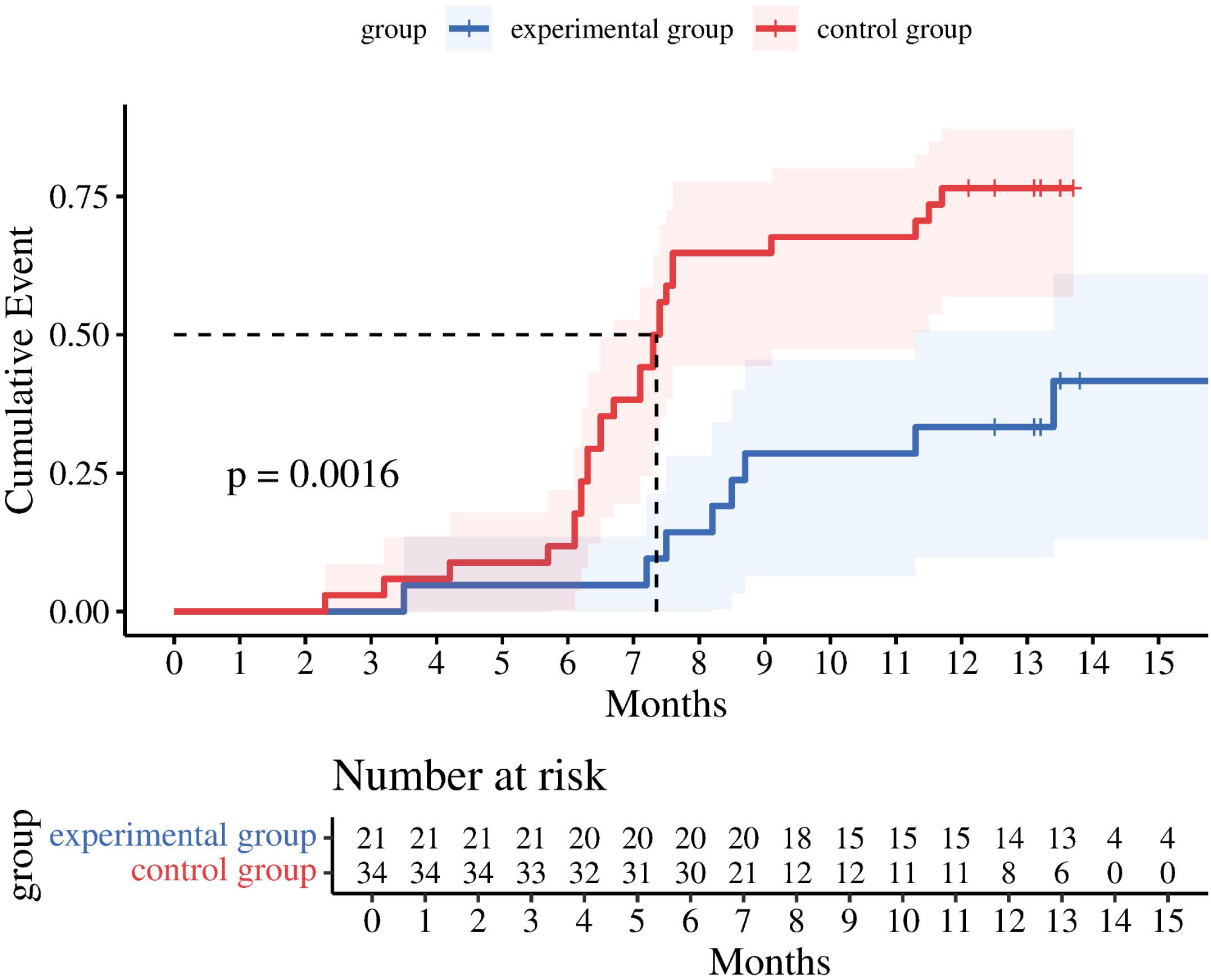
Kaplan–Meier analysis for PFS.

**Figure 2:**
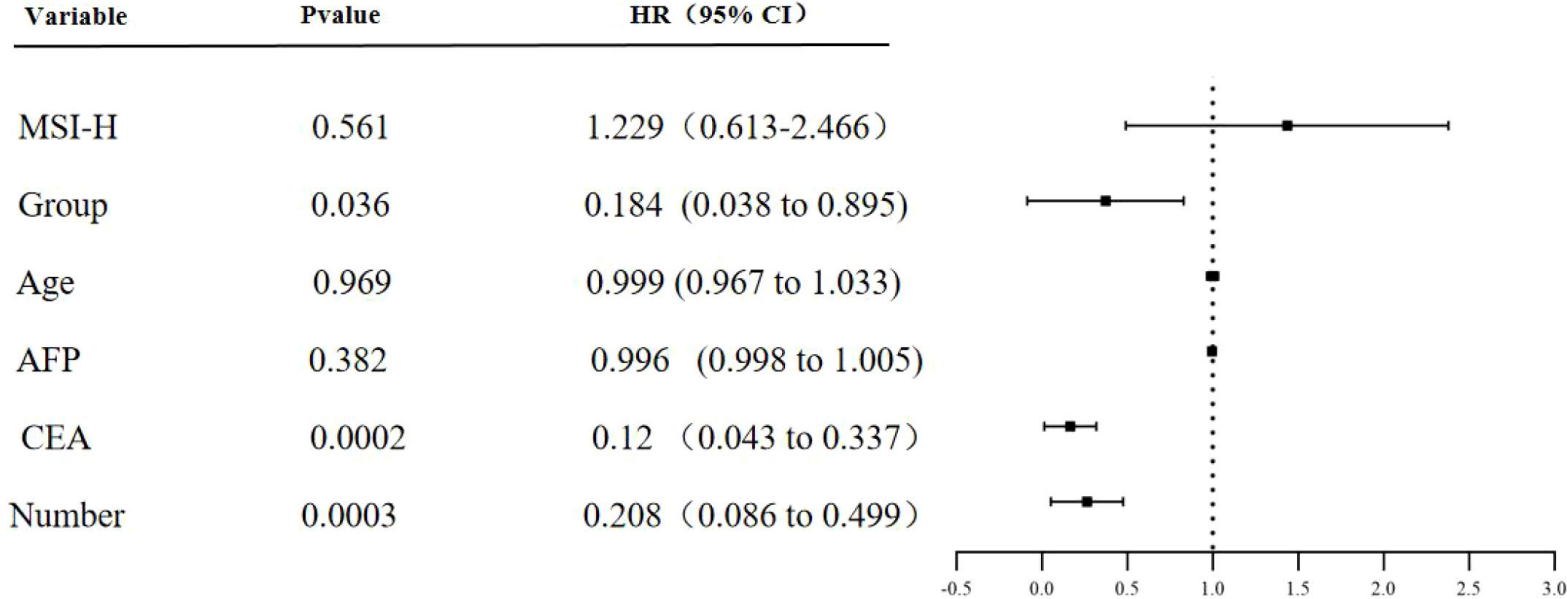
PFS multivariate analysis forest plot. AFP, alpha-fetal protein; CEA, carcinoembryonic antigen.

The one-year PFS rates were 66.7% and 23.5%. Log-rank test analysis (**Figure 1**) revealed a statistically significant difference in PFS between the two groups (P = 0.0016). The difference in PFS between the two groups was statistically significant (P = 0.0016). Multivariate Cox regression analysis was performed, incorporating variables including group assignment (experimental group=1, control group=2), age, MSI-H, AFP, CEA, tumor number. Analysis revealed statistically significant differences in patient grouping, number of liver metastases, and CEA levels between groups, while other factors showed no significant impact on PFS. Grouping showed a significant negative correlation with tumor recurrence (correlation coefficient = -1.692). After controlling for CEA, the experimental group still demonstrated a statistically significant prolongation of PFS, with a recurrence risk 0.184 times lower than the control group (P = 0.036). Normal CEA levels were negatively correlated with tumor recurrence (correlation coefficient -2.116), with patients having normal CEA levels exhibiting a recurrence risk 0.12 times lower than those with abnormal levels (P = 0.0002). The number of liver metastases was negatively correlated with tumor recurrence (correlation coefficient -1.573). Patients with a single liver metastasis had a recurrence risk 0.208 times lower than those with multiple liver metastases (P = 0.0003) (**Table 2**, **Figure 2**).

**Table 2:** Multivariate analysis of factors associated with PFS.

Variable	B	SEx	$\bar{x}$	df	P	HR	95% CI
Group	-1.692	0.806	4.402	1	0.036	0.184	0.038-0.895
Age (years)	-0.001	0.017	0.002	1	0.969	0.999	0.967-1.033
AFP	-0.004	0.004	0.766	1	0.382	0.996	0.988-1.005
CEA	-2.116	0.525	16.265	1	0.0002	0.12	0.043-0.337
MSI-H	0.207	0.355	0.338	1	0.561	1.229	0.613-2.466
Number of metastatic tumors	-1.573	0.448	12.332	1	0.0003	0.208	0.086-0.499

AFP, alpha-fetal protein; CEA, carcinoembryonic antigen.

### Kaplan–Meier analysis for PFS

The blue step curve represents the study subjects in the experimental group; the red step curve represents the study subjects in the control group. The blue shaded area is the 95% confidence interval for the cumulative event incidence rate in the experimental group. The red shaded area is the 95% confidence interval for the cumulative event incidence rate in the control group. The “+” marks on the curves in the figure represent censored data.

### Comparison of ORR, DCR, OS, and quality of life scores

**Figure 3:**
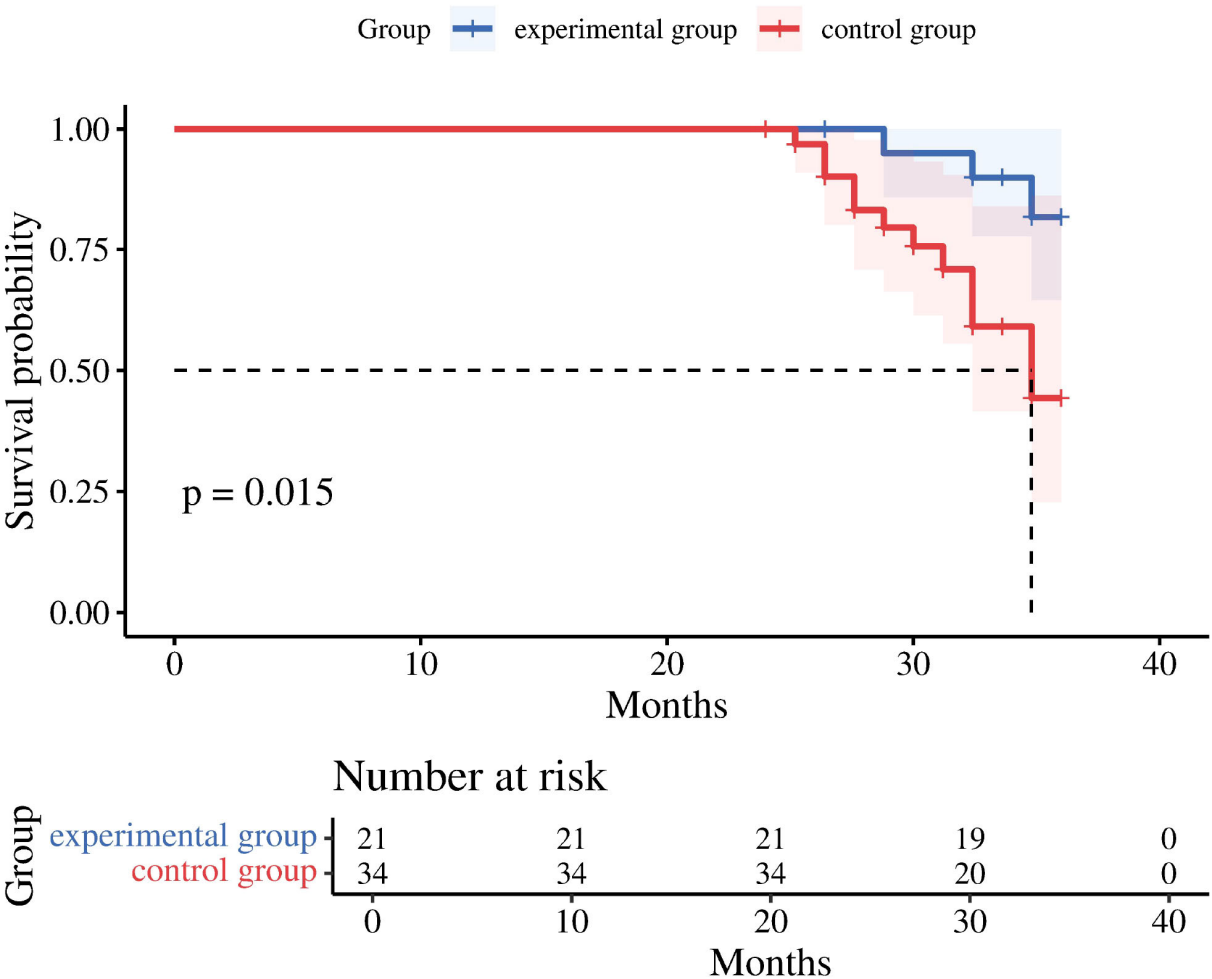
Three-year OS comparison chart.

Following completion of the full treatment course, investigators first assessed the ORR and DCR. Results showed ORR rates of 72.5% and 58.8% in the experimental and control groups, respectively, indicating a significantly higher ORR in the experimental group (P < 0.05). This suggests that combination therapy with tislelizumab and fruquintinib demonstrates superior efficacy compared to tislelizumab monotherapy in patients with advanced colorectal cancer liver metastases following MAW surgery. Although both groups demonstrated disease control rates exceeding 70%, no statistically significant difference was observed between them. Nevertheless, this suggests that combining targeted therapy with MAW may enhance disease control rates in patients with advanced colorectal cancer liver metastases (**Table 3**). In the subsequent first follow-up, the researchers conducted a quality-of-life assessment utilizing the EORTC Quality of Life Questionnaire QLQ-C30. Analysis of the symptom scales within this questionnaire indicates that higher scores correspond to more severe patient symptoms. The researchers observed that the median quality-of-life scores for both patient groups were identical and relatively elevated. This finding indirectly suggests that the side effects associated with immunotherapy and targeted drugs may significantly impact patients’ quality of life over time. The researchers conducted a 3-year follow-up, with survival assessments every 12 weeks (± 7 days). The study found that the experimental group demonstrated superior overall survival (OS) compared to the control group, with both groups exceeding 20 months of OS. This indicates that combining targeted and immunotherapy after microwave ablation for advanced colorectal cancer with liver metastases can effectively prolong survival. Patients receiving postoperative targeted therapy combined with immunotherapy demonstrated longer survival compared to those receiving tislelizumab monotherapy (P < 0.05) (**Figure 3**).This study conducted a 36-month follow-up. At the time of the final analysis, the experimental group (n = 21) experienced a total of 8 OS events, whereas the control group (n = 34) experienced 23 OS events. The median OS for the experimental group was not reached at the time of this analysis, with a 3-year OS rate of 62.4% (95% confidence interval: 41.1% - 82.7%). In contrast, the median OS for the control group was 29.4 months (95% confidence interval: 47.3% - 73.4%). These findings indicate that the experimental group sustained a significant overall survival advantage over the control group throughout the 3-year follow-up period.

**Table 3:** Comparison table of ORR, DCR and quality of life scores.

Variable	Experimental group		Control group	P	OR		95%CI
ORR				0.0483	2.24		0.664-7.55
effective	16		20				
Ineffective	6		14				
efficient	72.5%		58.8%				
DCR				0.53	1.53		0.405-5.77
Control	17		25				
Uncontrolled	4		9				
Control rate	80.9%		73.5%				
Quality of life Score				P		t	
Median (Min, Max)	72 (40, 90)	72 (45, 85)		>0.05		0.445	
OS(3 years)							
Median (Min, Max)	33.6 (26.4, 36)	30( 24, 36)		0.015			
Survival	13	11					
Death	8	23					
OS rate	62.4%	32.3%		<0.05			

### Three-year OS comparison chart

The blue step curve represents the study subjects in the experimental group; the red step curve represents the study subjects in the control group. The blue shaded area is the 95% confidence interval for the cumulative event incidence rate in the experimental group. The red shaded area is the 95% confidence interval for the cumulative event incidence rate in the control group. The “+” marks on the curves in the figure represent censored data.

### Safety

The incidence of adverse events in the observation group was 66.67%. No Grade 4–5 adverse events were reported. Common Grade 1–2 adverse events included pruritus and rash in 13 cases (61.9%), hypertension in 11 cases (42.4%), thrombocytopenia in 11 cases (42.4%), abdominal pain and diarrhea in 10 cases (47.6%), abnormal liver function in 9 cases (42.9%), decreased appetite in 6 cases (28.6%), and dry skin with ulceration in 5 cases (23.8%). Grade 3 adverse events included rash in 3 cases (14.3%), abdominal pain and diarrhea in 2 cases (9.5%), and thrombocytopenia in 1 case (4.7%). Symptoms improved after discontinuing symptomatic treatment for 3 weeks, and patients resumed the original treatment regimen (see **Table 4**).

**Table 4:** Adverse events.

Adverse events	Observation group(n=21)	
	Grade 1–2 AEs	≥ Grade 3 AEs
Abdominal pain, Diarrhea	61.9% (13)	14.3% (3)
Hypertension	52.4% (11)	4.7% (1)
Thrombocytopenia	52.4% (11)	0
Pruritus, Rash	47.6% (10)	9.5% (2)
Abnormal liver function	42.9% (9)	0
Decreased appetite	28.6% (6)	0
Dry or rupture skin	23.8% (5)	0

## Discussion

Despite substantial advancements in systemic therapies for colorectal cancer, the prognosis remains unfavorable, primarily due to consistently high rates of metastasis. Microwave ablation (MWA), a well-established local tumor ablation technique, induces coagulative necrosis in tumor tissue rapidly by employing high-frequency microwaves to agitate water and polar molecules within the tissue, thereby generating heat through friction. Compared to other ablation methods, such as radiofrequency ablation (RFA), MWA presents several advantages, including enhanced thermal efficiency, more extensive and uniform ablation coverage, and diminished susceptibility to the “heat sink effect.”

In recent years, a substantial body of research has extensively investigated the efficacy of MWA in the treatment of liver metastases. Within the context of colorectal cancer liver metastases, a comprehensive retrospective study demonstrated that MWA significantly decreased local recurrence rates, enhanced 5-year local progression-free survival, and reduced ablation time compared to surgical ablation. Multivariate analysis further corroborated these findings, indicating that the risk of local recurrence with RFA was 1.97 times greater than with MWA (12).A study investigating oligometastatic colorectal cancer found that image-guided thermal ablation techniques, such as radiofrequency and microwave ablation, offer long-term survival benefits comparable to those of surgical resection. The five-year overall survival rate for liver metastases ranges from approximately 43.1% to 76%, while for lung metastases, it is around 51%. Notably, when complete A0 ablation with margins greater than 5–10 mm is achieved, local control rates are comparable to those of surgical intervention, with the additional benefits of reduced trauma and quicker recovery. Moreover, when combined with chemotherapy, ablation significantly enhances the eight-year overall survival rate in patients with unresectable liver metastases, compared to chemotherapy alone (13, 14). Beyond its application in colorectal cancer, MWA exhibits significant potential in the treatment of liver metastases originating from various malignancies. A retrospective analysis spanning 26 years focused on liver metastases from breast cancer concluded that MWA, whether utilized as a standalone treatment or in conjunction with transarterial chemoembolization (TACE), results in favorable long-term survival outcomes. Specifically, the cohort receiving only MWA demonstrated a median survival of 5.6 years, with 1-, 3-, and 5-year survival rates of 89%. Conversely, the group treated with the combination of MWA and TACE exhibited a median survival of 2.4 years, with corresponding survival rates of 77%, 38%, and 22%. Both treatment modalities significantly outperformed laser-induced thermotherapy (LITT) and TACE as monotherapies (15). Additionally, MWA has been shown to effectively reduce tumor burden, manage local symptoms, and extend patient survival in cases of liver metastases from neuroendocrine tumors (16), gastric cancer (17), and pancreatic cancer (18). The synergistic effects of MWA in conjunction with immunotherapy are increasingly recognized as a promising area of research. Empirical evidence suggests that the integration of MWA with anti-PD-1/anti-CTLA-4 immunotherapy significantly enhances antitumor immunity, as evidenced by a marked prolongation of survival time in the combination therapy group compared to those receiving MWA alone or immune blockade therapies. In tumor re-challenge experiments, 66.7% (4 out of 6) of mice exhibited complete tumor rejection. Concurrently, there was a notable increase in intratumoral CD8^+^T cell infiltration, elevated CD4^+^and CD8^+^T cell counts in the spleen, and promotion of Th1-type immune response polarization. This was associated with significantly elevated concentrations of Th1 cytokines (IFN-γ, IL-18, IL-2) and reduced levels of Th2 cytokines (IL-4, IL-10) (19). Consequently, this study illustrates that the precise targeting of metastatic lesions by MWA not only achieves superior local control but may also modify the natural progression of the disease, potentially enabling patients to reach a no evidence of disease (NED) status and ultimately achieve long-term survival.

This study establishes that monotherapy with targeted agents following MWA surgery constitutes an independent risk factor for tumor recurrence when compared to the combined postoperative regimen of immunotherapy and targeted therapy. The median progression-free survival (PFS) for the experimental and control groups was 13.2 months and 7.35 months, respectively, as depicted in **Figure 1**. The vertical axis illustrates the cumulative probability of event occurrence. The overall survival curve for the experimental group was significantly lower than that of the control group, indicating a reduced probability of PFS occurrence in the experimental group. Multivariate Cox regression analysis further substantiated that the PFS incidence rate in the control group was 0.184 times higher than in the experimental group. Patients receiving the combination of targeted therapy and immunotherapy not only achieved extended PFS compared to those treated with tislelizumab alone but also exhibited a more substantial reduction in recurrence rates.A study has demonstrated that the combination of MWA and anti-PD-L1 therapy produces synergistic antitumor immune effects through a CXCL9-mediated mechanism in a murine model of colorectal cancer liver metastasis. This combination therapy significantly inhibited the growth of contralateral tumors, extended overall survival in mice, markedly increased the number of intratumoral CD8^+^T cells, enhanced their effector function, promoted TAM1 polarization, and upregulated CXCL9 expression in both TAM1 and tumor cells (20). Furthermore, a single-arm Phase II clinical trial reported favorable efficacy and safety outcomes in 12 patients with pMMR/MSS colorectal liver metastases treated with a PD-1 inhibitor in conjunction with chemotherapy and bevacizumab. The trial observed an ORR of 70% in primary tumors and 75% in liver metastases, with a DCR of 100%. Notably, three patients achieved a pathological complete response, and seven attained disease-free status. The median PFS was recorded at 9.2 months (21).In the treatment of advanced colorectal cancer with liver metastasis, the combination of molecularly targeted therapy and immunotherapy has shown superior efficacy compared to monotherapy. However, due to the limited sample size in this study, the multivariate Cox regression analysis did not reveal a significant correlation between microsatellite instability (MSI) typing and RAS gene typing.Multivariate Cox regression analysis has identified carcinoembryonic antigen (CEA) levels and the number of liver metastases as significant predictors of recurrence risk. Previous research suggests that in patients experiencing unexplained postoperative elevation of CEA following colorectal cancer surgery, FDG-PET exhibits a sensitivity of 79%–100% for detecting tumor recurrence (22, 23). Currently, the VEGFR inhibitor fruquintinib is extensively utilized as a third-line treatment for advanced colorectal cancer. Studies have demonstrated that among patients with baseline CEA levels exceeding 5 ng/L—an independent prognostic factor for overall survival—those treated with fruquintinib exhibited significantly improved median overall survival compared to those with baseline CEA levels above 5 ng/L (24). Consequently, we propose that abnormal CEA levels may serve as an early indicator of disease progression, particularly given that CEA predominantly originates from epithelial malignancies in colorectal cancer (25). MWA effectively eradicates liver metastases originating from solid tumors; however, it does not inhibit subsequent cancer cell proliferation nor prevent recurrence. Conversely, fruquintinib has been shown to effectively regulate CEA expression, thereby extending progression-free survival in patients with advanced colorectal cancer and exhibiting significant antitumor activity. A retrospective clinical study involving 810 patients identified the number of liver metastases as an independent predictor of overall survival following liver resection for colorectal cancer, with each additional metastatic lesion associated with a 6% increase in mortality risk (26). Furthermore, a randomized, double-blind, placebo-controlled Phase III clinical trial conducted in China demonstrated that fruquintinib, as compared to placebo, significantly prolonged overall survival in patients with advanced hepatocellular carcinoma who had progressed on or were intolerant to prior third-line chemotherapy (27). Consequently, for patients at risk of recurrence or mortality due to multiple liver metastases post-surgery, the combination of conventional PD-1 inhibitors with fruquintinib may constitute a promising strategy for enhancing survival in later lines of treatment.

The secondary outcome analysis indicated that patients in the experimental group exhibited significantly improved OS compared to those in the control group, with median OS surpassing 20 months in both groups. This suggests that the maintenance therapy regimen, which combines targeted therapy with immunotherapy following microwave ablation, is may effective in extending overall survival than tislelizumab monotherapy. In terms of tumor response, the ORR in the experimental group was 72.5%, significantly higher than the 58.8% observed in the control group, underscoring the superiority of the combined therapy in facilitating tumor reduction. DCR exceeded 70% in both cohorts; although the difference between groups was not statistically significant, it implies that postoperative combined targeted therapy may contribute to enhanced disease control. After a 36-month follow-up period, the study revealed that the 3-year OS rate of the experimental group was significantly higher than that of the control group. This suggests that the experimental group may continue to exhibit a notable overall survival advantage over the control group. In terms of patient-reported outcomes, the quality of life assessment, conducted using the symptom scales of the EORTC QLQ-C30 scale at the first follow-up, showed that the median scores of both groups were identical and relatively high. This finding implies that immune and targeted therapies may have certain side effects. Consequently, greater attention should be directed towards managing post-treatment complications in patients. Given that this study primarily concentrated on post-treatment complications, only the symptom scales were analyzed. To gain a comprehensive understanding of patients’ quality of life, a multi-dimensional analysis is warranted. A comprehensive single-center retrospective study has demonstrated that ultrasound-guided percutaneous microwave ablation for liver metastases originating from colorectal cancer is both safe and effective, achieving a complete ablation rate of 99.27% at the lesion level and 97.81% at the patient level. The rate of local tumor progression was observed to be 5.35% at the lesion level and 16.06% at the patient level. Independent risk factors for local progression included a tumor diameter of ≥3 cm, proximity to major blood vessels, proximity to the diaphragm, and a lack of response to preoperative chemotherapy (28). The failure to respond to preoperative chemotherapy is an independent risk factor for local progression, necessitating postoperative immunotherapy or targeted therapy. Early research identified that the tumor-associated protein B7-H1 (PD-L1) is overexpressed in various human cancers, facilitating immune evasion by inducing antigen-specific T-cell apoptosis through receptors other than PD-1 (29). In a multicenter Phase II clinical trial (NECTAR), the combination of tislelizumab (a PD-1 inhibitor) and long-course chemoradiotherapy in patients with locally advanced rectal cancer resulted in a 40.0% pathological complete response rate, with a 56.0% incidence of treatment-related adverse events (30). Fruquintinib has been shown to selectively inhibit VEGFR-1, 2, 3 tyrosine kinases, effectively blocking the VEGF signaling pathway and inhibiting tumor angiogenesis. This leads to significant tumor growth suppression in colon cancer xenograft models and patient-derived xenograft models, with enhanced efficacy when combined with chemotherapy agents like oxaliplatin (31).

The international multicenter Phase III study (FRESCO-2) demonstrated that in patients with metastatic colorectal cancer who had undergone a median of four prior lines of therapy and were refractory to trifluorouracil-temozolomide, regorafenib, or both, treatment with fruquintinib significantly enhanced overall survival and progression-free survival compared to placebo, achieving a disease control rate of 56% (32, 33). A recent open-label, single-arm Phase II trial indicated that the combination of fecal microbiota transplantation (FMT) with tislelizumab and fruquintinib as a third-line or subsequent treatment regimen significantly improved survival outcomes in patients with refractory microsatellite stable (MSS) metastatic colorectal cancer. The median PFS was 9.6 months, the OS was 13.7 months, the ORR was 20%, and the disease control rate was 95%. This treatment regimen demonstrated a manageable safety profile, with no treatment-related fatalities reported (34). Although microwave ablation can temporarily achieve a tumor-free state, its recurrence rate remains high. The postoperative administration of PD-1 inhibitors may prevent immune escape.In conjunction with the anti-angiogenic agent fruquintinib, which functions by inhibiting tumor formation and preventing immune escape, this dual-faceted strategy markedly extends PFS and diminishes disease recurrence in patients with advanced colorectal cancer liver metastases.

Concerning safety, although the incidence of adverse events during treatment was not negligible, only Grade 1–3 adverse reactions were observed. These reactions were within manageable limits and were well tolerated by patients. Notably, thrombocytopenia occurred in over 50% of cases as an adverse reaction, underscoring the necessity of preemptive measures to prevent thrombocytopenia prior to administration in order to avert subsequent bone marrow suppression reactions.This study possesses several limitations. Firstly, as a prospective investigation, it necessitates long-term follow-up. However, due to various constraints, the study was limited to a 3-year follow-up period, which restricts the assessment of long-term efficacy. Secondly, the sample size in this study is relatively limited, which may diminish the statistical power of multivariate analyses, thereby impacting the stability and generalizability of the research findings. Additionally, a small sample size may elevate the risk of not adequately controlling for potential confounding variables. Consequently, the results of this study should be considered as exploratory and preliminary, with their validity and reliability requiring further confirmation through future multicenter, large-sample confirmatory studies. In subsequent research, the scientific rigor and generalizability of the conclusions can be enhanced by increasing the sample size, extending the recruitment period, and engaging in multicenter collaboration. This study investigated the efficacy of combining fucoxitinib and tislelizumab following microwave ablation in the treatment of metastatic colorectal cancer (mCRC). The control group received only PD-1 inhibitor monotherapy, which deviates from the standard first-line or subsequent treatment regimens typically employed for mCRC in contemporary clinical practice. The findings of this study are particularly relevant for patients experiencing multiple complications during first-line treatment post-microwave ablation, especially those unable to tolerate standard chemotherapy due to compromised physical health and severe comorbidities.(4)In conclusion, the integration of MWA, PD-1 inhibitors, and anti-angiogenic agents may offer superior outcomes compared to conventional treatment regimens for the systemic management of mCRC.

## Data Availability

The original contributions presented in the study are included in the article/supplementary material. Further inquiries can be directed to the corresponding author.
